# Universal School Meals and Associations with Student Participation, Attendance, Academic Performance, Diet Quality, Food Security, and Body Mass Index: A Systematic Review

**DOI:** 10.3390/nu13030911

**Published:** 2021-03-11

**Authors:** Juliana F. W. Cohen, Amelie A. Hecht, Gabriella M. McLoughlin, Lindsey Turner, Marlene B. Schwartz

**Affiliations:** 1 Department of Nutrition and Public Health, Merrimack College, 315 Turnpike Street, North Andover, MA 01845, USA; 2 Department of Nutrition, Harvard T.H. Chan School of Public Health, 677 Huntington Ave, Boston, MA 02115, USA; 3 Institute for Research on Poverty, University of Wisconsin-Madison, 1180 Observatory Drive, Madison, WI 53706, USA; aahecht2@wisc.edu; 4 Implementation Science Center for Cancer Control and Prevention Research Center, Brown School, Washington University in St. Louis, St. Louis, MO 63130, USA; gmcloughlin@wustl.edu; 5 Division of Public Health Sciences, Department of Surgery, Washington University School of Medicine, Washington University in St. Louis, St. Louis, MO 63130, USA; 6 College of Education, Boise State University, 1910 University Drive, Boise, ID 83725-1742, USA; lindseyturner1@boisestate.edu; 7 Rudd Center for Food Policy and Obesity, Department of Human Development and Family Sciences, University of Connecticut, 1 Constitution Plaza, Suite 600, Hartford, CT 06103, USA; marlene.schwartz@uconn.edu

**Keywords:** universal school meals, nutrition, community eligibility provision, breakfast, lunch, attendance, academic performance, BMI

## Abstract

The school environment plays an important role in children’s diets and overall health, and policies for universal free school meals have the potential to contribute to positive child health outcomes. This systematic review evaluates studies examining the association between universal free school meals and students’ school meal participation rates, diets, attendance, academic performance, and Body Mass Index (BMI), as well as school finances. The search was conducted in accordance with the Preferred Reporting Items for Systematic Review and Meta-Analyses (PRISMA). A search for studies published in economically developed countries published through December 2020 was performed in PubMed, Education Resources Information Center (ERIC), Thomson Reuters’ Web of Science, and Academic Search Ultimate, followed by examining the references in the resultant literature. A total of 47 studies were identified and the Newcastle-Ottawa Scale (NOS) was applied to assess bias. Nearly all studies examining universal free school meals found positive associations with school meal participation. Most studies examining universal free school meals that included free lunch found positive associations with diet quality, food security, and academic performance; however, the findings of studies examining only universal free breakfast were mixed. Research findings were similarly mixed when examining attendance as an outcome. Concerns about adverse outcomes on student BMI were not supported by the literature; in fact, several studies detected a potentially protective effect of universal free school meals on BMI. Research examining the impact of universal free meals on school finances was limited, but suggest that lower-income school districts in the U.S. may have positive financial outcomes from participation in universal free school meal provisions. Additionally, providing free meals to students may be associated with improved household incomes, particularly among lower-income families with children. Further research is needed to examine the financial implications of universal free meals for both school districts and families. Overall, universal free school meals may have multiple benefits for students and countries should consider universal free school meal provisions with strong nutrition guidelines. (PROSPERO registration: CRD42020221782).

## 1. Introduction

Globally, schools are recognized as an important setting to promote healthy behaviors, as children typically spend a substantial amount of their waking hours at school [1,2,3]. As children’s eating preferences often persist into adulthood, schools can provide meaningful opportunities to promote and establish healthier diets through access to nutritious foods, including breakfast and lunch [1,4,5,6,7]. Among countries with developed economies, school meals are a common feature of national safety net programs and typically provide students with breakfast and/or lunch with a focus on improving diet quality, particularly among low-income populations [1]. Many countries have nutrition standards for school meals, which can have important short- and long-term health implications for children, including promoting optimal growth and cognitive development and reducing the risk of food insecurity and obesity [8,9,10,11,12,13,14,15,16,17,18]. School meal programs are commonly means-tested (e.g., based on family income, eligibility for the Supplemental Nutrition Assistance Program [SNAP], etc.), whereby access to subsidized free or reduced-price meals are available to lower-income students. However, policies that support universal free school meals (i.e., meals provided at no cost to all children who wish to participate) are gaining attention as a strategy to reduce stigma and diet-related disparities among lower-income students, promote children’s nutrition more broadly (school meals are often healthier than meals brought from home), and potentially lower administrative costs for schools [19,20,21,22,23,24,25,26,27,28]. 

Despite the potential benefits of universal free school meals, they are currently only available in a small number of countries. Beginning in 1943, Finland was the first country to offer universal free school meals, and Sweden implemented them two years later in 1945 [29,30]. More recently, Estonia began providing free school meals to all students in 2002, and South Korea implemented a universal free school meal program in 2011 [11,31]. Additionally, England implemented the Universal Infant Free School Meals (UIFSM) policy for state-funded schools in 2014 and Scotland introduced a similar policy in 2015, both of which only apply to children in their first three years of primary school (children ages 4–7 years at the beginning of the school year) [10,32]. Japan’s school meal program, which was first implemented in 1947, is universal but not free; participation in the school meal program is mandatory, and low-income families receive financial support for school meals from the local and/or national government [33,34].

In the United States, there have been multiple provisions supported by the USDA that have enabled high-poverty schools to provide universal free school breakfast and/or lunch. These include: Provision 1, Provision 2, Provision 3, and most recently, the Community Eligibility Provision (CEP) which was introduced as part of the Healthy Hunger-Free Kids Act of 2010 and became available to eligible schools nationwide in 2014 (see Supplemental Table S1 for a summary of school meal provisions in the United States) [8,35]. To be eligible to opt into CEP, ≥40% of students in a district or school must be from low-income households, which is determined using existing administrative data (e.g., participation in SNAP). However, in the 2019–2020 school year, only about 69% of eligible schools in the U.S. were participating in CEP, in part due to concerns about insufficient reimbursement from the federal government [36,37].

While proponents of universal free school meals cite numerous benefits, including higher school meal participation rates and improved student diet quality, academic performance, and attendance, concerns have also been raised about the potential detrimental impact on students’ body mass index (BMI) and/or school finances [38,39]. To date, the research examining universal free school meals has not been systematically evaluated. A commentary by Hecht and colleagues, which included a non-systematic review of CEP in the United States, concluded that there was growing evidence of an association with positive health and academic benefits for children, but that more research was needed [40]. A better understanding of how universal free school meals impact students and schools can help inform school meal policies globally. Therefore, the aim of this study was to systematically review the international evidence regarding the impact of universal free school meals on students’ school meal participation rates, diets, attendance, academic performance, and body mass index (BMI), and as well as school finances.

## 2. Materials and Methods

This review was conducted according to the Preferred Reporting Items for Systematic Reviews and Meta-Analysis (PRISMA) guidelines [41]. The protocol was registered with the PROSPERO International Prospective Register of Systematic Review prior to data extraction (protocol registration number: CRD42020221782) [42].

### 2.1. Data Sources and Search Strategies

Four online databases were used: PubMed, Education Resources Information Center (ERIC), Thomson Reuters’ Web of Science, and Academic Search Ultimate. The search strategy was comprised of combinations of the following keywords (adapted for each database): school, universal, free, community eligibility, provision, reimbursement, access, poverty, hunger, meal, breakfast, lunch. Articles in English published since the start of the literature through December 2020 were reviewed. To identify additional potentially relevant studies, a search of the reference lists of these articles as well as a review of all articles citing the resultant literature (using Google Scholar) was performed.

### 2.2. Study Selection

Eligible studies were quantitative research articles evaluating universal free school meals and school meal participation rates, academic performance, attendance, Body Mass Index (BMI), diet quality, food insecurity, and/or school finances. Our inclusion criteria were English, peer-reviewed publications or official government reports within countries with developed economies (i.e., members of the Organization for Economic Co-Operation and Development [OECD]) [43], and conducted in primary (i.e., elementary) and secondary (i.e., middle, and/or high) schools during the academic year (terminology throughout reflects how grade levels are referred to within each country). Studies from Japan examining universal school meals (mandatory for all students but not free) were also included due to the similar nature of the school meal program compared with universal free school meal programs and thus research findings were considered relevant and informative. The following types of articles were excluded: non-English articles; qualitative research; articles that did not examine universal free school breakfast or lunch (e.g., snacks, milk programs, or afterschool programs); initiatives that occurred outside of the school year (i.e., holiday or summer vacations); studies conducted in non-OECD countries; and articles that only compared across different universal free school meal models (i.e., universal free breakfast offered in the cafeteria versus universal free breakfast provided in the classroom). Two independent reviewers conducted the searches and screened titles and abstracts, and the lead author (JFWC) screened full texts. Articles with unclear eligibility were reviewed by the research team. A total of 47 studies that met the inclusion criteria were identified and included in this paper (*n* = 25 studies conducted in the U.S. and *n* = 22 studies conducted in other OECD countries). Due to the heterogeneity of the study designs and outcome measures of the included studies, the data could not be combined to reanalyze as meta-analyses. Therefore, a qualitative narrative review was used to synthesize the results of the included studies.

### 2.3. Quality Assessment

Assessment of study quality and biases were based on adapted Newcastle–Ottawa Scales (NOS) for cross-sectional and cohort studies, which are commonly used to assess non-randomized research [44,45,46]. Each study was assessed by two authors using the following criteria: selection, comparability, and outcome. Quality assessments were interpreted based on the following categories: very high risk of bias (0–3 points), high risk of bias (4–6 points), and low risk of bias (≥7 points) [47]. Quality Assessments are presented in Supplemental Table S2 (cross-sectional studies) and Supplemental Table S3 (cohort and quasi-experimental studies).

## 3. Results

**Figure 1:**
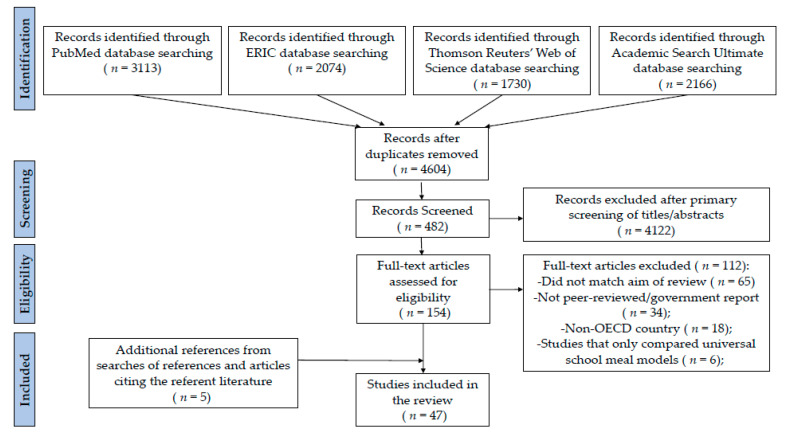
Flow chart for systematic review.

The original search of the four databases (PubMed, ERIC, Thomson Reuters’ Web of Science, and Academic Search Ultimate) identified a total of 9083 articles. After removing duplicates, 4604 articles remained. The primary screening excluded 4122 records. The full text of the remaining 156 publications were assessed in detail, and 121 articles were excluded. The primary reasons for exclusion in this step concerned the study objectives (i.e., did not aim to evaluate universal free school meals or only compared universal free school meal models), publication type (i.e., grey literature), and the location where the study was conducted (i.e., non-OECD countries). Five additional articles were identified from references or publications citing the referent literature. Therefore, a total of 47 articles were included in this review (Figure 1). As seen in Supplemental Tables S2 and S3, quality scores for the included articles ranged from 3 (very high risk of bias) to 10 (low risk of bias). Over half of the included studies (*n* = 26) were classified as having a low risk of bias while *n* = 12 articles had high risk and *n* = 10 articles had very high risk (one study had both high and low risk of bias for differing outcomes assessed). Research conducted in the U.S. is presented separately from other OECD countries in each section of the review due to the large number of studies and the unique nature of universal free school meal provisions in the U.S. (i.e., universal free school meals are primarily available only in lower-income schools).

### 3.1. School Meal Participation

A primary goal of universal free school meals is to increase school meal participation. Of the 11 peer-reviewed publications and four government reports conducted in the U.S. that examined school meal participation as an outcome (*n* = 15 studies; Table 1), 14 found a positive association between universal free school meals and National School Lunch Program (NSLP) or School Breakfast Program (SBP) participation and one found mixed results. One peer-reviewed study and two government reports examining universal free school meals in the United Kingdom (UK) also found positive associations with school meal participation (Table 2). Among the studies conducted using methodology with a low risk of bias, all 11 found positive associations between universal free school meals and school meal participation rates.

In the United States, several studies have examined the impact of universal free SBP and program participation. In a study conducted by Leos-Ubel et al. in New York City among elementary and middle school students, access to universal free breakfast was positively associated with SBP participation, with rates varying by student socio-economic status (SES) [48]. Among students with the lowest household income levels who were already eligible for free school meals, there was an increase in participation by 5% (*p* < 0.05). Among students previously eligible for reduced-price meals, there was a 21% increase in SBP participation (*p* < 0.01), and among students eligible for full- price meals previously, there was a 36% increase in participation (*p* < 0.01). Similar results were observed in a study conducted by Ribar and colleagues in a school district in North Carolina where three elementary schools removed universal free breakfast while one school introduced it [49]. Overall, universal free breakfast was associated with 16.4% higher rates of SBP participation (*p* < 0.05), with the greatest increase in participation rates among students from higher-income households who were not previously eligible for free or reduced-price meals (27.5%; *p* < 0.05). Nearly identical results were observed in a government report examining the pilot of universal free breakfast in six school districts in six states, with schools within each district randomly assigned to universal free breakfast or control (means-tested based on eligibility for free or reduced-price meals); after one year, universal free breakfast was associated with a 16 percentage point increase in participation (*p* = 0.01), again, with the largest increases observed among students not previously eligible for free or reduced-price meals [50]. In a second government report examining the extended impact of this pilot program, the increases in participation were maintained over three years [51]. Another study conducted by Soldavini and associates in North Carolina elementary, middle, and high schools found that providing universal free breakfasts was positively associated with significantly greater odds of breakfast participation at all grade levels, with the exception of high school students who were previously eligible for free or reduced-price meals [52]. A third study by Wahlstrom et al. examining six elementary schools in Minnesota also observed high SBP participation rates among schools with universal free breakfast, although no statistical analyses were conducted and this study was assessed to have a very high risk of bias [22]. In a cross-sectional study conducted by Khan and colleagues in a Vermont middle school serving universal free breakfast, food-insecure students were significantly less likely to eat breakfast at home compared with food-secure students, and nearly all (91.3%) of the food-insecure students reported eating free breakfast at school [53]. However, a cross-sectional study in Philadelphia, Pennsylvania conducted by Dykstra et al. found that students participated in the free SBP only 31% of the time (however, changes in participation rates were not measured) [54]. Although there were significantly higher participation rates among minority students compared with their white peers, there were no differences in participation by eligibility for free or reduced-price meals or by food-insecurity levels.

Several studies have also examined universal free school meal provisions prior to the implementation of CEP in the U.S. One study in Texas conducted by Rivas examining a school district that implemented Provision 2 observed a 16% increase in overall school meal participation rates (although this was not analyzed for statistically significant differences and the study was considered to have a very high risk of bias) [55]. Similarly, in a study conducted by Schwartz et al. among middle schools in New York City, Provision 2 was positively associated with NSLP participation, with differences observed by student SES; on average there was an 11% increase in NSLP participation among students not previously eligible for school meals (*p* < 0.05) and a 5.4% increase among students from lower-income households (*p* < 0.01) [39]. A government report examining a no-fee school meal pilot program in several states found an increase in lunch participation by approximately 10 percentage points (although this study was deemed to have a very high risk of bias) [56]. Another government report evaluating an initiative to eliminate reduced-price fees for school meals observed similar changes, with an 11% increase in lunch participation and 9% increase in breakfast participation among students previously eligible for reduced-price meals (and this study also was assessed to have a very high risk of bias) [57].

The CEP was introduced in 2010, and several studies tested its impact using pre-post designs. Pokorney et al. examined all CEP schools (*n* = 654) before and after implementation compared with eligible non-CEP schools (i.e., schools that chose not to participate in CEP but met the eligibility criteria [*n* = 1221]) in Pennsylvania and found that on average CEP was associated with an 8% increase in school lunch participation [58]. In sub-analyses examining participation by student SES, NSLP participation rates were higher among students not previously eligible for free or reduced-price meals, but slightly lower among lower-income students in CEP schools compared with non-CEP schools. Turner and colleagues also examined the introduction of CEP, as well as the use of Provisions 1, 2, and 3 in public schools in California, and found that universal free school meals were associated with a 5.8 percentage point increase in NSLP participation and 3.5 percentage point increase in SBP participation [59]. Lastly, Tan et al. used national data from K-8 schools to address this question and found that CEP was associated with an 11.7% higher likelihood of participating in the NSLP among near-cutoff students (i.e., students who were near the cutoff and had uncertain eligibility for free or reduced-price meals [*p* = 0.023]) and a 19% higher likelihood of participation among students previously eligible for full-price meals (*p* < 0.001) [60]. CEP was also associated with a 19.6% higher likelihood of participation in the SBP among students previously eligible for full-price meals in this study (*p* < 0.05).

Among research conducted in the UK, a government report examining the pilot of Universal Infant Free School Meals (UIFSM) in England among primary school students in three local authorities found that universal free school meals were associated with approximately a 30% increase in the percent of students taking a school lunch at least once a week (i.e., roughly 90% of students had a school lunch at least once week in schools with universal free school meals compared with 60% of students in matched control schools) [61]. The greatest increases in participation were among students who were not previously eligible for school meals. Similarly, in another government report examining universal free school meals in Scotland, school lunch participation increased by 22 percentage points (from 53% to 75%), with the greatest increases among students not previously registered for free school meals (an increase of 28 percentage points) [62]. Another study conducted in primary schools in Scotland examining universal free school meals also found the greatest increases in lunch participation were among students not previously eligible for free school meals (14.4 percentage point increase [*p* < 0.001]) [63].

To summarize, there is a large literature examining the relationship between offering universal free meals and student participation. Overall, the findings consistently show that student participation in school meal programs increases when meals are provided at no costs. Further, the increase in participation tends to be largest among students who previously did not qualify for free or reduced-price meals.

### 3.2. Diet Quality and Food Insecurity

Another priority of universal free school meals is to improve the nutritional quality of children’s diets and reduce food insecurity. In the presence of strong nutrition standards, school meals may improve children’s diets directly by providing healthy foods (i.e., fruits, vegetables, whole grains, etc.) [23,24,25,26,28,64]. There may be indirect benefits as well; prior research has found that healthy school meal consumption is associated with decreased intake of less healthy foods outside of school, potentially attributable to increased satiety from nutrient dense, high fiber school meals [65]. Additionally, when school meals are provided at no cost, families are able to save those funds and increase their purchasing power for other foods, further reducing food insecurity. Of the six peer-reviewed publications and one government report conducted in the U.S. that examined diet-related outcomes (*n* = 7 studies [Table 1]), two found a positive association between universal free school meals and dietary quality and two found a positive association with food security. Three studies examined only universal free breakfast and found mixed results with diet quality. Among the 19 studies conducted in other OECD countries (18 peer-reviewed and one government report [Table 2]), including the UK, Denmark, Norway, Japan, Greece, and New Zealand, 13 found improvements in students’ dietary outcomes and three found no association. Of the three studies that examined food insecurity, two studies found improvements and one found no association. Of the studies examining dietary outcomes that were considered to have a low risk of bias, the majority (6 out of 7) found improvements in dietary outcomes. All three studies examining food insecurity had a low risk of bias. 

Among studies examining diet quality in the United States, four studies examined universal free breakfasts and one examined CEP. Crepinsek and colleagues examined a national sample of elementary schools participating in universal free breakfast versus matched control schools offering traditional (means-tested) breakfasts [66]. This study found that universal free breakfast was positively associated with the consumption of a nutritionally substantive breakfast, including more servings of fruit and dairy. However, there was no association with breakfast skipping or overall dietary intakes over a 24-h period. Similarly, a government report examining the pilot implementation of universal free school breakfasts in six states found no association with nutrients consumed over the course of a day, but students with universal free breakfast were more likely to eat breakfast on all five school days (*p* < 0.01) [50]. A study by Dykstra et al. using a national sample of schools also found that when universal free breakfast was provided, rates of breakfast skipping remained comparable between food-secure and food-insecure students [54]. A study in Boston conducted by Kleinman examined the diets of students and found that those with improved nutrient intakes had significant increases in SBP participation [67]. However, this was not observed among all students receiving school breakfasts (i.e., higher SBP participation was observed among the subsample of students with improved diets, but not all SBP participants had observed improvements in their diets). One cross-sectional study without a comparison group examining universal free school meals in six CEP elementary schools in Virginia found that participation in school meals was associated with consumption levels within recommendations by the Dietary Guidelines for Americans, with students consuming roughly 2.5% out of the recommended limit of 10% of daily calories for added sugars (although consumption was not compared with rates prior to implementing CEP) [68].

Two studies examining food insecurity in the U.S. found that CEP was associated with improvements in food security. Poblacion and colleagues used simulation modeling with national data and estimated that CEP would lead to a 3.73% increase in students becoming food secure and a 3.17% increase in food security among previously food-insecure households with children [69]. Similarly, a study by Gross et al. conducted in Maryland found that when compared with students at CEP schools, students attending schools in another district that opted not to participate in CEP had increased odds of being in a household that was food insecure (OR 2.85, 95% CI 1.67, 4.88) [19].

When examining just universal free breakfast, a study conducted in Norway among 10th grade students found that free breakfast was associated with overall higher Healthy Eating Index (HEI) scores among male students, but no significant change was observed among female students (and this study was considered to have a very high risk of bias) [70]. Additionally, two studies in Wales examined the same dataset with slightly different analyses, and both found that free school breakfasts were associated with an increase in the number of healthy items eaten at breakfast [71,72], with larger increases observed in lower-income schools [72]. Overall, there was no association between free breakfasts and breakfast skipping [71], but in sub-analyses, free breakfast was inversely associated with breakfast skipping among students from lower-income households and among students at schools serving higher proportions of students with household poverty [72]. Examining 24-h recalls among a subsample of the students participating in the Welsh study, researchers found that among children receiving a free school breakfast, nearly half also had a breakfast at home prior to coming to school, but this was not associated with significant differences in calories consumed over a 24-h period [73].

When examining school lunch initiatives in Norway, a country where nutrition standards are voluntary, studies measuring students’ diets were mixed. One study by Ask et al. examining 9th grade students participating in a pilot free lunch intervention for four months found no association with healthy food scores (although this study was considered to have a very high risk of bias) [74]. A study by IlløKken et al. assessed students ages 10–12 in an intervention school offering free school lunch (compared with a control group comprised of students at the same school and an additional control school) and found that free school lunch was positively associated with healthy food scores after six months of exposure, primarily due to increased consumption of fruits, vegetables, and fish spread [75]. In a separate report of results after following the same students for a year, Vik et al. found that healthy food scores remained significantly higher in the intervention group compared with the control group [76], but there were no significant changes in overall meal frequency (e.g., frequency of eating breakfast, lunch, or dinner on weekdays) [77].

Results were similarly mixed among studies conducted in England. One study conducted by Spence et al. examining UIFSM in two primary schools found that free school lunches had lower consumption of non-milk extrinsic sugar (i.e., added sugar) and biscuits (i.e., shortbread cookies) at lunch, but higher intake of cakes/sweet puddings (i.e., desserts), which were offered daily with school lunches after the main meal was served [78]. The reductions in added sugar were also observed among the students’ overall diets (i.e., over the course of an entire day). Another government report by Kitchen and colleagues examining UIFSM found no association with student diets [61], and a third conducted by Gatenby and associates examining a free lunch scheme similarly found no association with overall nutrients consumed throughout the day (although this study was deemed to have a very high risk of bias). Notably, students who received school lunches consumed less at lunch than students who brought their lunches from home, but then compensated by eating more foods outside of the lunch period [79].

In other countries, studies examining school meal programs have generally found positive results concerning dietary quality. One study in Japan examining the mandatory school lunches served in elementary and junior high schools found that consuming school lunches was positively associated with total diet quality [34]. Additionally, a study conducted in the greater Tokyo area among children ages 6–12 found that providing universal (mandatory) school lunches was associated with a reduction in SES-related dietary disparities, particularly regarding fruit and vegetable consumption [80]. A smaller study conducted in one kindergarten class in New Zealand found that offering free lunches to all students in the class was associated with significant reductions in snack food consumption while at school (although this study was assessed to have a very high risk of bias) [81]. In the OPUS School Meal study, which provided free lunches to all 3rd and 4th grade students in nine schools in Demark for three months (and the students ate packed lunches from home for three months) found significant improvements in students’ diets, including 16% higher vegetable intakes (*p* < 0.0001) and 48% higher fish intakes (*p* < 0.0001), as well as 30% lower intakes of saturated fats (*p* < 0.0001) [82]. Lastly, a similar study conducted in Denmark among four schools that provided free school lunches for two months (compared with control schools with lunches packed from home only) also found that free meals were associated with improved dietary quality, including reductions in saturated fat and snacks and increases in vegetables and fish [83]. When the school meals were no longer provided at no cost after the two-month period, participation in school lunch became limited and there were no longer improvements in dietary quality observed.

One study in New Zealand examined universal free breakfast and food insecurity. Mhurchu et al. conducted a study in 14 primary schools in New Zealand with universal free breakfast and found a significant decrease in children’s self-reported short-term hunger, but no association with overall food insecurity levels [84]. In contrast, two studies conducted in Greece (as part of the ‘DIATROFI’ program) with universal free lunches found significant reductions in food insecurity, with the greatest decreases among food-insecure households with hunger [85,86].

### 3.3. Attendance

Researchers have theorized that universal free school meals could increase students’ school attendance rates through two mechanisms. The first explanation is that students from low-income households may be motivated to attend school to access the food available. Secondly, improved nutrition from school meals may also decrease the incidence of illness, which could improve attendance. Of the seven peer-reviewed publications and two government reports conducted in the U.S. that examined attendance (*n* = 9 studies [Table 1]), five found a positive association between universal free school meals and attendance (primarily among sub-populations) and four found no association. A limited number of studies (*n* = 3 peer-reviewed publications) have been conducted in other OECD countries, which included Denmark and New Zealand; none found an association with overall attendance and one found a positive association among students with higher school breakfast participation rates in sub-analyses (Table 2). Among the studies considered to have a low risk of bias, half (5 out of 10) found positive associations with attendance.

When examining studies only implementing universal free breakfast, Bartfeld et al. conducted a study in approximately 1000 elementary schools in Wisconsin with varying breakfast models (including universal free and means-tested programs) and found that for lower-income students only, universal free breakfast was associated with an increase in the percent of days attended (0.24 percentage point increase; *p* = 0.023) and a decrease in the percent of students with low attendance (3.5 percentage point decrease; *p* < 0.001) [87]. Similarly, in the Kleinman et al. study in Boston (*n* = 97 students), improvements in attendance were observed only among the students who improved their nutritional status (which was associated with participation in the SBP) [67]. Leos-Urbel and colleagues also observed improvements in attendance among subpopulations within New York City elementary and middle schools; universal free breakfast was associated with a small increase in attendance among low-income Black students and higher-income Asian students [48]. In the study conducted by Ribar et al. in North Carolina among elementary schools that stopped offering universal free breakfasts, no impact on attendance was observed [49]. Lastly, two government reports examining the pilot of the universal free school breakfast program in six states found no association with attendance after either one year [50] or three years [51] of exposure.

Two studies examined universal free school meals and attendance. Gordanier et al. evaluated 3–8th grade students throughout South Carolina and found CEP was inversely associated with absences (i.e., improved attendance) among elementary students but not middle school students [88]. Bartfeld and colleagues examined elementary schools throughout Wisconsin and found that after two years of exposure, there were no associations between CEP and overall attendance rates, but a 3.5 percentage point reduction was observed in the percent of low-income students with low attendance (*p* = 0.045) [89]. It was also noteworthy that no associations with attendance were observed within the first year of implementation. Similarly, Schwartz et al. found no association between Provision 2 and overall attendance rates among middle school students in New York City [39].

Among other OECD countries, in the study conducted by Mhurchu et al. in the 14 schools with a free school breakfast program, as well as in the study by Munday and colleagues examining free lunches among a kindergarten classroom (both conducted in New Zealand), neither found an association with overall attendance rates (although the study by Munday et al. was considered to have a very high risk of bias) [81,84]. However, in secondary analyses examining students with higher school breakfast participation (attendance at least 50% of the time at school breakfast), Mhurchu et al. found small but significant improvements in attendance (1.6% increase; *p* = 0.016). Laursen and associates also found no association between a free school lunch program and overall attendance among 3rd and 4th grade students in Denmark [90]. However, most of the students in these studies had limited exposure to free meals (ranging from 2.5 to 10 months).

### 3.4. Academic Performance

Academic performance may also be influenced by universal free school meals, both directly through potential improvements in nutrition, as well as indirectly through potential increases in school attendance rates [17,91,92,93,94]. Among the ten studies in the U.S. (*n* = 8 peer-reviewed and *n* = 2 government reports [Table 1]), all three examining CEP found positive associations with academic performance, while the six studies examining universal free breakfast were mixed. Similarly, one government-funded project conducted in England found a positive association between universal free school meals and academic performance while three studies (conducted in New Zealand and Wales) found no association when examining universal free breakfast (*n* = 4 studies [Table 2]). Specifically among the studies with a low risk of bias, 3 out of 7 found a positive association with academic performance. 

Of the studies examining universal free breakfast, Kleinman et al.’s study in Boston found significant improvements in academic performance (i.e., math test scores), but only among students who improved their nutrient intakes [67]. Similarly, Walhstrom and colleagues observed positive trends in standardized achievement test scores within six elementary schools (compared with three control schools) in Minnesota after piloting universal free breakfasts, although no statistical analyses were conducted and this study was considered to have a very high risk of bias [22]. In Bartfeld et al.’s study in Winsonsin elementary schools, universal free breakfast was positively associated with math (0.07 SD higher, *p* = 0.001) and reading (0.04 SD higher, *p* = 0.035) test scores, but only among higher income students [87]. Contrasting with those studies, Leos-Ubel et al.’s study among elementary and middle school students in New York City [48], Ribar et al.’s study in North Carolina [49], and the two government report examining the pilot of universal free breakfast found no significant associations with test scores after one or three years of exposure [50,51].

Among studies examining school meal provisions that include lunch in the U.S., Gordanier et al.’s study among 3–8th grade students in South Carolina found that CEP was positively associated with math test scores (0.06 standard deviation increase) among elementary students, but did not have any significant associations with reading scores in elementary school, nor any test scores among middle school students [88]. Similarly, Schwartz et al. found a positive association between Provision 2 and academic performance among middle school students in New York City; math and English Language Arts standardized test scores were significantly higher, with the greatest improvements observed among higher-income students [39]. Lastly, in a study conducted by Taylor and colleagues in Vermont, CEP was associated with higher perceptions of improved academic performance and readiness to learn according to school staff surveys, although no direct, objective measurements of academic performance were collected (and the study was considered to have a very high risk of bias) [95].

In studies conducted in other OECD countries (i.e., New Zealand and the UK), Mhurchu et al. found no association between a free school breakfast program and academic performance in New Zealand primary schools [84]. Similarly, no association was found within primary schools in Wales between implementation of a free breakfast initiative and cognitive scores among two studies examining the same data using slightly different analyses [71,72]. However, in a government report examining UIFSM, free lunch was associated with improved academic performance; students made on average 4–8 weeks more progress compared with similar students in control schools [61]. 

### 3.5. Body Mass Index

One concern about universal free school meals has been the potential adverse impact on children’s BMI, particularly if a child receives a breakfast at home and then receives a second breakfast and lunch at school [38,39]. However, if healthier meals are provided by schools, they may reduce the risk of obesity to the extent that they replace less nutritionally balanced foods with higher quality school meals [96,97]. The evidence that school meals are on average healthier than lunches brought from home supports this theory [25,27]. Further, it may be that increased satiety from healthy lunch consumption leads to reduced consumption of less healthy foods after school [65]. To date, only one peer-reviewed study and one government report in the U.S. have examined BMI and universal free school meals (*n* = 2 studies [Table 1]), and five studies (*n* = 4 peer-reviewed publications and *n* = 1 government report [Table 2]) have been conducted in other OECD countries including England, Norway, and the Netherlands. The majority (6 out of 7) found either no association with BMI or a reduced probability of developing overweight and obesity. Among the limited number of studies considered to have a low risk of bias, 2 out of 3 found universal free school meals were associated with lower BMIs among students.

In the U.S., Schwartz and colleagues found no association between free lunches offered through Provision 2 and BMI among middle school students in New York City [39]. In sub-analyses, Provision 2 was associated with a 2.5% *reduced* probability (*p* < 0.01) of obesity among higher-income students. In a government report examining the pilot of universal free breakfast, there was no association with the prevalence of overweight after one year of exposure to free school breakfasts [50].

A study in the Netherlands conducted by Bartelink et al. found that providing children ages 4–12 with a free lunch (in additional to some structured physical activity after lunch) was inversely associated with BMI z-score after two years of follow-up (standardized effect size = −0.083, *p =* 0.01) [98]. In a study conducted by Ask et al. among 10th graders in Norway, providing free breakfasts also appeared to be protective against excess weight gain; BMI remained unchanged among students receiving free school breakfast, whereas there was a significant increase in the BMI of students in the control group over the span of four months (although this study was deemed to have a very high risk of bias) [70]. Another study by Ask et al. conducted in Norway among 9th grade students found no association between a pilot free lunch program and BMI after four months of exposure, and this study also was considered to have a very high risk of bias [74]. Similarly a government report in England found no association between UIFSM and BMI [61]. These results contrast with a study conducted in Norway that found a free school lunch program was associated with higher BMI z-scores, compared with a decrease in BMI z-scores among the control students (F = 10.007, *p* = 0.002), despite the healthier food scores observed among the students with free lunch [76].

### 3.6. Finances

Concerns have also been raised about the impact of universal free school meals on school finances, specifically due to the increase in costs from preparing and serving more meals [38,39]. However, 5 out of the 6 peer-reviewed publications and government reports in the U.S. reported that increased food and labor costs were balanced by increased revenues from school meals served through federal reimbursements. Of note, these estimates of the cost of implementation do not include the increase in cost to the U.S. federal government. (Table 1). Additionally, one study in Scotland estimated the overall costs of providing universal free lunches (Table 2). Among these studies, only one was considered to have a low risk of bias, and this study found positive outcomes for school finances with universal free school meals.

In the U.S., a study in Texas that examined a school district with Provision 2 found that implementation was associated with a 5% increase in annual revenue (statistical significance was not assessed and the study was assessed to have a very high risk of bias) [55]. Similarly, a government report examining the pilot of universal free school breakfasts found that there was an increase in labor costs, but these were more than offset by the increase in meals served (and thus an increase in federal reimbursements), resulting in an average savings of USD 0.11 per breakfast served for districts [50]. Another government report examining the USDA No-Fee School Meal Pilot Program in three states found that providing universal free school meals was associated with a 33% increase in federal reimbursement overall and reduced administrative costs [56]. In a third government report examining CEP, this provision was associated with increased federal meal reimbursements (increase of 5.6% for NSLP and 1.9% for SBP) [37]. Staff also spent significantly less time distributing and processing applications for free and reduced-price meals and/or verifying student eligibility, which resulted in an average savings of 68 min per student annually (translating to a labor-saving cost of approximately USD 29 per student per year). This time savings is partially offset by an average 30 min/student increase in staff time annually to claim reimbursable meals (*p* < 0.01). Overall, this government report found that federal funding via reimbursements per student increased by 13.5% (or USD 5.33 per student annually), resulting in potential net gains for school districts participating in CEP. Similarly, in a study conducted in Vermont, school staff (e.g., principals, food service staff, and business managers) perceptions were measured using an online survey, and roughly half of participants perceived that school finances had improved (52.4%), while only 44% perceived that the school meal program deficit was reduced [95]. No objective measurements of school finances were collected in this study. Conversely, a government report examining schools that eliminated reduced-price fees within 5 states found that federal reimbursements for the school meals served only partially offset the program costs with higher participation rates (although this report was deemed to have a very high risk of bias) [57]. In Scotland, a government report examining school meals found that the costs of preparing meals varied (from £1.79 to £4.65 per meal). The cost per meal decreased as the number of meals served increased, likely due in part to economies of scale (e.g., buying in bulk or through contract negotiations) [62].

**Table 1:** Characteristics of studies conducted in the United States included in the systematic review.

Author, Year	Location; Participant Characteristics	Study Design	Year(s)	Universal Meal Provision	Outcome Measure(s)	Results	Risk of Bias ^2^
Adams et al. 2020 [68]	Virginia; 6 Title I elementary schools (grades 1–5), *n* = 1155 plate waste measurements	CS	2016	CEP	Diet: Added sugar consumption at lunch (measured using plate waste determined using digital photography)	In CEP schools, foods selected had on average 11.2 g of added sugar and beverages had on average 11.0 g of added sugar. Students consumed on average 6.6 g of added sugar from foods and 3.6 g of added sugar from beverages (~10% of calories consumed from foods and ~35% calories consumed from beverages; ~2.5% of added sugars consumed out of the 10% recommend by the Dietary Guidelines for Americans)	Low
Bartfeld et al. 2019 [87]	Wisconsin; elementary schools throughout the state with varying breakfast models (including universal free and mean-tested)	QE: Pre/post (with control)	2009–2010 to 2013–2014 SY	USBP	(1) Attendance: percent of school days attended and low attendance (i.e., attending fewer than 95% of available days) measured among student in grades 1–5 (2) Academic Performance: test scores in reading and math measured among student in grades 3–5	(1) USBP was not associated with attendance overall, but in sub-analyses was associated with increased attendance among low-income students; USBP was associated with a 0.24% pt ↑ in the % of days attended (*p* = 0.023) and a 3.5% pt ↓ in the percent of students with low attendance (*p* <0.001) (2) USBP was not associated with academic performance overall, but in sub-analyses was associated with 0.07 SD high math scores (*p* = 0.001) and 0.04 SD higher reading scores (*p* = 0.035) among higher-income students	Low
Bartfeld et al. 2020 [89]	Wisconsin; 37 CEP elementary schools and 108 comparison (i.e., eligible non-CEP) elementary schools (grade 1–5)	QE: Pre/post (with control)	2013–2014 to 2015–2016 SY	CEP	Attendance: percent of school days attended and low attendance (i.e., attending fewer than 95% of available days)	After two years of exposure, CEP was associated in a 3.5% pt ↓ in low attendance (*p* = 0.045) compared with control schools. In sub-analyses, CEP was associated with a 4.2% pt ↓ in the probability of low attendance (*p* = 0.035) among lower-income students.	Low
Bernstein et al. 2004 [51] (USDA Report)	USA; Six school districts (in six states); elementary schools within each district randomly assigned to USBP (*n* = 79 schools) or control (maintain means-tested SBP; *n* = 74 schools).	QE: Pre/post (with control)	1999–2000 to 2002–2003 SY	USBP	(1) Participation (2) Attendance (3) Academic Performance: test scores in reading and math	(1) Offering free school breakfasts was associated with an ↑ in breakfast participation that was maintained for three years (a 15% pt gain after three years; *p* = 0.01). (2) No association with attendance (3) No association with test scores	Low
Brown 2009 [57] (GAO Report)	USA; 5 states and 14 districts in other states that implemented ERP	QE: Pre/post (no control)	2007–2008 SY	ERP	(1) Participation (2) Finances	(1) ERP was associated with an ↑ in participation in the SBP (9% average increase) and NSLP (11% average increase) among students who were eligible for reduced-price meals (2) Federal reimbursements only partially offset programs costs for the participation states/school districts	Very High
Crepinsek et al. 2006 [66]	USA; national sample of elementary schools (153 matched schools in six school districts with USBP or means-tested breakfast [*n* = 4358 students, grades 2–6])	Cluster RCT	1999–2000 to 2002–2003	USBP	Diet: food and nutrient intakes (measured using one 24-h recall)	USBP was positively associated with the consumption of a nutritionally substantive breakfast (80% vs. 76%; *p* < 0.01), including increased servings of fruit and dairy. There was no association with overall breakfast skipping or overall dietary intakes over a 24-h period.	Low
Dykstra et al. 2016 [54]	Philadelphia, PA; 16 schools (students grade 4–6; *n* = 821 student/parent dyads) with USBP	CS	2013	USBP	(1) Participation (2) Diet: Breakfast skipping (measured using the Breakfast Patterns Survey [student self-report])	(1) On the day of data collection, 38.8% of students reported consuming a school breakfast and participating in the SBP on 32.1% of possible days (with 87.0% of students participating in the SBP at least 1 day during the fall semester). There was significantly higher participation among minority students (Black students participated on 36.5% of days, Hispanic students participated on 25.0% of days, and white students participated on 18.7% of days (*p* < 0.001). No differences in SBP participation by free or reduced-price eligibility or by food insecurity levels. (2) 16.9% of students reported skipping breakfast on the morning of data collection. Rates of skipping breakfast did not differ between students from food-insecure households and students from food-secure households.	High
Gordanier et al. 2020 [88]	South Carolina; elementary and middle schools throughout the state that adopted CEP vs. non-CEP schools (both eligible and non-eligible schools), students grade 3–8	QE: Pre/post (with control)	2013–2014 to 2015–2016 SY	CEP	(1) Attendance (2) Academic Performance: State standardized test scores (Math and English Language Acquisition]	(1) CEP was associated with a ↓ in absences among elementary students (−0.231 days per year; *p* < 0.05). No significant associations were observed with absences among middle school students. (2) CEP was associated with an ↑ in math test scores among elementary students (0.06 SD; *p* < 0.01). No significant associations were observed for math scores among middle school students nor for reading scores for elementary or middle school students.	Low
Gross et al. 2019 [19]	Maryland; One district with 5 CEP schools and one matched control district with 3 schools (CEP-eligible but not participating), *n* = 427 household surveys	CS	2017	CEP	Diet: Food insecurity (measured using the USDA Six-Item Short Form of the Food Security Survey Module [parent report]	CEP was associated reduced odds of household food insecurity (i.e., students had twice the odds of being in a food-insecure household if they attended a school that was CEP-eligible but not participating [OR 2.85, 95% CI 1.67, 4.88]).	High
Khan et al. 2011 [53]	Vermont; one middle school (grades 6–8) with USBP, *n* = 373 students	CS	2005	USBP	Diet: Food insecurity (measured using a 9-item validated survey [student self-report])	Food insecure children were significantly less likely to eat breakfast at home compared with food secure children (32.9% vs. 18.6% of students did not eat breakfast at home; *p* = 0.007), and 91.3% of food insecure students reported eating breakfast at school.	High
Kleinman et al. 2002 [67]	Boston, Massachusetts; three schools before and after implementing USBP (*n* = 97 students in grades 4–6)	QE: Pre/post (no control)	1998–1999 to 1999–2000 SY	USBP	(1) Diet: Nutrient intakes and hunger (measured using 24-h dietary recalls and 5-item version of the Child Hunger Index Child Report survey [student self-report] + an 8-item hunger/food insufficiency questionnaire [parent report]) (2) Attendance (3) Academic Performance: test scores in math, reading, science, and social studies (based on school records)	(1) USBP was not associated with differences in the percent of students who were nutritionally at risk overall, but children who had improvements in nutritional status had significant ↑ in USBP participation (*p* < 0.001) and ↓ in self-reported hunger (mean change in hunger score of −1.4; *p* < 0.0001). (2) Among students with improved nutrient intakes, there was a significant ↓ in absences (−4.4 days absent; *p* < 0.01). (3) Among students with improved nutrient intakes, there was a positive association with math grades (mean change 0.6; *p* < 0.05). No other significant associations were observed with grades.	High
Leos-Urbel et al. 2013 [48]	New York City, New York; elementary and middle schools before and after implementing USBP (*n* = 723,843 students in grades 3–8)	QE: Pre/post (no control)	2002–2003 to 2003–2004 SY	USBP	(1) Participation (2) Attendance (3) Academic performance: scores in statewide English and math tests	(1) USBP was associated with ↑ breakfast participation (an increase of 5% among students previously eligible for free meals [*p* < 0.05])., 21% among students previously eligible for reduced price meals [*p* < 0.01], and 36% among students previously eligible for full-price meals [*p* < 0.01]). (2) There was no association with overall attendance rates. In sub-analyses, universal free breakfast was associated with a small ↑ in attendance among low-income black students (0.37%; *p* < 0.01) and higher-income Asian students (0.25%; *p* < 0.05). (3) There was no association with academic performance.	Low
Logan et al. 2014 [37] (Report to USDA)	National; 7 states (285 participating LEAs ^1^ and 528 matched non-participating LEAs)	QE: Pre/post (with control)	2009–2010 to 2012–2013 SY	CEP	(1) Participation (2) Finances	(1) CEP was associated with ↑ participation (5.2% increase in NSLP participation, *p* < 0.01; 9.4% increase in SBP participation, *p* < 0.01) (2) CEP was associated with ↑ federal reimbursement (5.6% for NSLP [3.5% pts, *p* < 0.01]; 1.9% for SBP [3.5% pts, *p* < 0.01]). CEP was also associated with ↓ in time spent by staff distributing/processing applications for FRP meals (*p* < 0.01) and verifying eligibility of students (*p* < 0.01), resulting in a combined savings of on average 68 min/student annually, which represents a labor saving cost of approximately USD 29/student annually (partially offset by increases in staff time to claim reimbursable meals [increase of 30 min/student annually, *p* < 0.01]. Federal funding (reimbursements) per student ↑ by 13.5% (USD 5.33/student annually *p* < 0.01), and there was no impact on non-Federal finances (e.g., state reimbursement or student payments for non-reimbursable meals) resulting in potential net gains for LEAs participating in CEP.	Low
McLaughlin et al. 2002 [50] (USDA Report)	USA; Six schools districts (in six states); elementary schools within each district randomly assigned to USBP (*n* = 79 schools) or control (maintain means-tested SBP; *n* = 74 schools).	Cluster RCT	1999–2000 to 2000–2001 SY	USBP	(1) Participation (2) Diet: Frequency of breakfast and nutrients consumed (measured by 24 h recall [with parent assistance]) (3) Attendance (4) Academic Performance: test scores for math and reading and cognitive tests (5) BMI: objective measurements at school (6) Finances	(1) USBP was associated with 16% pt ↑ in participation (*p* = 0.01), with the largest increases among students not previously eligible for free or reduced-price breakfasts. (2) Students in schools with USBP were more likely to eat breakfast on all five school days (*p* < 0.01) but there was no difference observed in most nutrients consumed over the course of a day. (3) There were no differences observed in attendance. (4) There were no differences observed in math or reading score gains nor cognitive functioning. (5) There were no differences observed in the prevalence of overweight. (6) The increases in breakfast participation resulted in lower per-meal labor costs in schools with USBP, with the increases in labor costs offset by the increase in meals served (average cost per breakfast served was USD 0.11 lower).	High/ Low^2^
Poblacion et al. 2017 [69]	USA; national dataset of households with children and school meal participation rates to model potential impact of CEP	SM	2014	CEP	Diet: food insecurity (measured using simulation modeling based on change in income-to-poverty ratios of food-insecure people in households with children using prevalence estimates from national data)	Free lunches from CEP was associated with an estimated increase of 3.73% of students becoming food secure (due to families increasing their food purchasing power). When examining the combined impact of USBP and NSLP with CEP, the estimated increase in purchasing power was associated with 3.23% of food insecure households with children becoming food secure.	Low
Pokorney et al. 2019 [58]	Pennsylvania; all CEP schools (*n* = 654) and eligible non-CEP schools (*n* = 1221)	QE: Pre/post (with control)	2013–2014 to 2014–2015 SY	CEP	Participation	CEP was associated with an 8% ↑ in lunch served (RR = 1.08, 95% CI 1.03, 1.12). In sub-analyses, CEP was associated with an 69% ↑ in lunches served among higher-income students (RR = 1.69, 95% CI 1.11, 2.56), but also a slight decrease among students previously eligible for free or reduced-price meals (RR = 0.91, 95% CI 0.86, 0.96).	Low
Ribar et al. 2013 [49]	North Carolina; elementary schools that changed between USBP and mean-tested SBP (*n* = 4 schools) and matched schools with no change (*n* = 6)	QE: Pre/post (with control)	2007–2008 to 2008–2009 SY	USBP	(1) Participation: Grades 1–5 (2) Attendance: Grades 1–5 (3) Academic Performance: state standardized test scores in math and reading (grades 3–5) and science (grade 5),	(1) USBP was associated with a 16.4% ↑ in breakfast participation overall (*p* <0.05), with the greatest increases among higher-income students (27.5%; *p* < 0.05). (2) No association with attendance. (3) No association with test scores.	Low
Rivas 1994 [55]	Brownsville, Texas; one school district before and after implementing Provision 2	QE: Pre/post (no control)	1993–1994 SY	Provision 2	(1) Participation (2) Finances	(1) Provision 2 was associated with a 16% ↑ in overall school meal participation (2) Provision 2 was associated with a 5% ↑ in district food service revenue	Very High
Robinson 1994 [56] (GAO Report)	USA; 3 states with four school districts implementing the USDA No-Fee School Meal Pilot Program	QE: Pre/post (no control)	1990–1991 to 1992–1993 SY		(1) Participation (2) Finances	(1) Universal free meals was associated with ↑ participation (10% pt for NSLP). (2) Federal reimbursement increased by 33% overall due to increased student meal participation; districts incurred reduced administrative costs.	Very High
Schwartz et al. 2020 [39]	New York City, New York; middle schools with universal free lunch through Provision 2 (free breakfast was available in all schools prior to the start of the study)	QE: Pre/post (no control)	2010–2013	Provision 2	(1) Participation (2) Attendance (3) Academic Performance: standardized test scores (English Language Arts [ELA] and math) (4) BMI: objective measurements by schools	(1) Provision 2 was associated with ↑ school lunch participation (5.39% among lower-income students [*p* < 0.01] and 10.97% among higher income students [*p* < 0.05]). (2) No association with attendance (3) Provision 2 was associated with ↑ in math scores (0.036 SD; *p* < 0.01) and ELA scores (0.030 SD; *p* < 0.01), with the greatest increases among higher-income students (0.083 SD [*p* < 0.01] in math and 0.059 SD [*p* < 0.01] for ELA). (4) No association with BMI. In sub-analyses, Provision 2 was associated with a 2.5% reduced probability (*p* < 0.01) of obesity among higher-income students.	Low
Soldavini et al. 2019 [52]	North Carolina; 2285 public schools (elementary, middle, and high schools) with varying SBP models (including USBP)	CS	2017	USBP	Participation	USBP was positively associated with the odds of student participation at breakfast for all school levels, except high school students who were previously eligible for free or reduced-price meals.	Low
Tan et al. 2020 [60]	USA; national data from K-8 schools (80 CEP schools [*n* = 842 students] and 118 non-CEP schools [*n* = 1463 students])	QE: post-only (with control)	2013–2015	CEP	Participation	CEP was associated with ↑ NSLP participation among students near the cutoff for free or reduced-price meals (11.7% higher likelihood of participation, *p* = 0.023) and higher-income students (eligible for full- price) [18.5% higher likelihood of participation, *p* < 0.001] compared with students at schools not participating in CEP. CEP was also associated with ↑ USBP participation among higher-income students (19.6%; *p* < 0.05).	Low
Taylor et al. 2020 [95]	Vermont; 116 school staff members (e.g., principals, food service staff, business managers, and nurses) from K-12 schools throughout the state with CEP	CS	2017	CEP	(1) Academic performance: staff perceptions (measured using an online survey) (2) Finances: staff perceptions (measured using an online survey)	(1) Within CEP schools, a higher percentage of school staff perceived that free meals were associated with improved academic performance (64.4% agreed vs. 34.5% disagreed) and students were more ready to learn (83.0% agreed vs. 14.8% disagreed). (2) Approximately half of participants (52.4%) perceived that school finances had improved with CEP, but only 44% perceived that the school meal program deficit was reduced.	Very High
Turner et al. 2019 [59]	California; Public schools throughout the state with varying school meal provisions	QE: Pre/post (with control)	2013–2014 to 2016–2017 SY	CEP or Provision 1, 2, or 3	Participation	Universal free school meals was associated with ↑ lunch participation (5.79% pt increase) and ↑ breakfast participation (3.48% pt increase)	Low
Wahlstrom et al. 1999 [22]	Minnesota; 6 elementary schools piloting USBP and 3 control schools	QE: Pre/post (no control)	1993–1994 to 1996–1997	USBP	(1) Participation: students in grades K-8 (varying by school) (2) Academic Performance: standardized achievement test scores (grades 3–6)	(1) High school breakfast participation rates were observed and maintained in schools with USBP (no statistical analyses conducted). (2) A general increase in standardized achievement test scores for math and reading were observed (no statistical analyses conducted).	Very High

CS: Cross-sectional study; GAO: Government Accountability Office; LEA: Local Education Agency; QE: Quasi-experimental; RCT: Randomized controlled trial; SM: Simulation modeling; SFA: School Food Authority; USBP: Universal School Breakfast Program. ^1^ LEAs include traditional school districts as well as public and non-public nonprofit local entities (e.g., charter schools, non-public schools, archdiocese running multiple non-public schools, etc.) that enter into agree-ments with State agencies to operate the NSLP and SBP. ^2^ Risk of Bias varied by outcome (see Supplemental Table S3).

**Table 2:** Characteristics of studies conducted in other (non-U.S.) OECD countries included in the systematic review.

Author, Year	Location; Participant Characteristics	Study Design	Year(s)	Universal Meal Provision	Outcome Measure(s)	Results	Risk of Bias ^1^
Andersen et al. 2014 [82]	Denmark; 9 schools (3–4th grade students) assigned to free lunch (3 months) and packed lunch from home (3 months); *n* = 834 students	QE: Pre/post (with control)	2011–2012 SY	Free school lunch (+ snacks)	Diet: Foods and nutrients consumed (measured using a validated 7-day food record tool [self-administered]	Free school lunches was associated with improved diets, including higher intakes of, vegetables (16% higher intake; *p* < 0·0001) and fish (48% higher; *p* < 0·0001, which resulted in higher intakes of vitamin D (42% higher; *p* < 0·0001) and iodine (11% higher; *p* < 0·0001). Additionally, students consumed significantly less saturated fat (30% lower; *p* < 0·0001). There were no significant differences in total calories.	Low
Asakura et al. 2017 [34]	Japan; 14 elementary schools (*n* = 629 students) and 13 junior high schools (*n* = 281 students)	CS	2014	Universal school lunches	Diet: Diet records completed by parents/guardians on three non-consecutive days (two school days and one weekend day) + plate waste at school	School lunches were positively associated with total diet quality (the prevalence of inadequate nutrient intakes was higher on weekend days compared with school days for almost all of the nutrients assessed).	High
Ask et al. 2006 [70]	Norway; 10th grade students in one school with 1 intervention classroom with free breakfasts for 4 months (*n* = 26 students) and 1 control classroom (*n* = 28 students)	QE: Pre/post (with control)	2005	Pilot free breakfast intervention	(1) Diet: Diet quality (measured using a non-validated FFQ, which was used to calculate overall HEI scores) (2) BMI: objective measurements by school nurse	(1) Free breakfast was positively associated with overall HEI scores among male students (16 pt increase in HEI score; *p* < 0.05) (2) No changes in BMI were observed among students with free breakfast, but was significantly higher among control students who did not receive a school breakfast	Very High
Ask et al. 2010 [74]	Norway; 9th grade students (1 intervention school with free lunches for 4 months [*n* = 58 students] and 1 control school [*n* = 92 students])	QE: Pre/post (with control)	2007	Pilot free lunch intervention	(1) Diet: Healthy food scores (measured using a non-validated FFQ,) (2) BMI: objective measurements by research team	(1) No association with food scores (2) No association with BMI	Very High
Bartelink et al. 2019 [98]	Netherlands; 4 intervention and 4 control schools (*n* = 1676 children age 4–12 years).	QE: Pre/post (with control)	2015–2017	Free school lunch (+ structured PA after lunch)	BMI: objective measurements by research team	Free school lunch ( + PA) was associated with ↓ BMI z-score after two years of follow-up (standardized effect size = −0.083, *p =* 0.01)	Low
Dalma et al. 2020 [85]	Greece; 28 intervention (*n*= 1442 students) and 23 control primary schools (*n*= 986 students)	Cluster RCT	2014–2015 SY	Free lunch (+ nutrition education)	Diet: Food insecurity (measured using the Food Security Survey Module [FSSM]; parent report)	Free school lunch was associated with ↓ food insecurity (average FSSM score decrease of 0.31 points; *p* = 0.045), with the greatest reduction observed among food insecure households with hunger (average decrease of 1.04 points; *p* = 0.023).	Low
Gatenby 2011 [79]	England; two primary schools (one higher- and one lower-income [147 students ages 8–11, and a subsample of *n* = 20 students with food diaries])	CS	2004	Universal free lunch	Diet: Plate waste + Food diaries (5 days) and photos taken by students	Students who received school meals consumed significantly less at lunch on average compared with students who brought meals from home. However, due to compensation outside of lunch, there were no differences in overall nutrients consumed throughout the day.	Very High
Holford 2015 [63]	Scotland; all primary schools (students ages 4–11 years)	QE: Pre/post (with control)	2003–2013	Universal free school lunch	Participation	Universal free school lunch was associated with an ↑ on participation among students previously eligible for free school meals (3.3% pt; *p* < 0.05) as well as among students not previously eligible (14.4% pt; *p* < 0.001)	Low
IlløKken et al. 2017 [75]	Norway; one intervention elementary school with students receiving free school lunch for six months (*n* = 55 students) and one control school (*n* = 109 students); students ages 10–12 years	QE: Pre/post (with control)	2014–2015 SY	Free school lunch	Diet: Healthy food scores (measured using an FFQ)	Free school lunch was associated with ↑ in healthy food scores (change in total healthy food score of 1.7 vs. 0.5; *p* < 0.01), primarily due to an increased frequency of consuming fruits (*p* < 0.01), vegetables (*p* < 0.01), and fish spread (*p* = 0.02).	High
Jenkins et al. 2015 [73]	Wales; 111 primary schools randomly assigned to free school breakfast (*n* = 55 schools) or control (delayed intervention [*n* = 56 schools]); students ages 9–11 years	Cluster RCT	2004–2005 to 2006–2007 SY	Primary School Free Breakfast Initiative	Diet: 24-h recalls	There were no differences in the nutritional quality of breakfasts consumed at school or at home, except significantly higher levels of selenium (5.1 μg vs. 3.2 μg; *p* < 0.01 and carbohydrates (59.8 g vs. 48.7 g; *p* < 0.01) in school meals. Among students who ate a school breakfast, 49% had already consumed a breakfast at home that morning, although there were no significant differences in caloric intake over a 24-h period.	High
Laursen et al. 2015 [90]	Denmark; 9 schools (3–4th grade students) assigned to free lunch (3 months) and packed lunch from home (3 months); *n* = 797 students	Cluster RCT	2011–2012 SY	Free school lunch (+ snacks)	Attendance	No association with attendance rates	Low
MacLardie et al. 2008 (Scottish Govt report) [62]	Scotland; 5 local authorities	QE: Pre/post (no control)	2007–2008 SY	Free school meals trial for P1-P3 pupils (universal free lunch)	(1) Participation (2) Finances	(1) An ↑ in participation of 22% pts was observed in schools with free school lunches, with the greatest increases among students not previously registered for free school meals (28% pts). An increase in participation was also observed among students previously eligible for free school meals (4% pt increase). (2) The cost of implemented school meals varied from £1.79 to £4.65 per additional meal. Costs tended to be lower in areas with a greater number of additional meals served.	Very High
Mhurchu et al. 2012 [84]	New Zealand; 14 primary schools with staggered implementation of free school breakfasts (*n* = 424 students ages 5–13 years)	Cluster RCT	2010	Free school breakfast program	(1) Diet: Short-term hunger (measured using satiety scale for children [self-report]), student breakfast habits (parent-report), and child/household food security (measured using the CCHIP Scale [parent-report]). (2) Attendance (3) Academic Performance: standardized tests of math and literacy	(1) Free school breakfast was associated with a ↓ in children’s self-reported short-term hunger (increase of 8.6 units on the satiety scale; *p =* 0.001). No association with child or household food security, or breakfast frequency. (2) No association with overall attendance. In a sub-analysis examining students who frequently attended the SBP (≥50% of the time), free school breakfast was associated with a 1.6% increase in attendance (*p* = 0.016). (3) No association with academic performance	Low
Moore et al. 2014 [72]	Wales; 111 primary schools randomly assigned to free school breakfast (*n* = 55 schools) or control (delayed intervention [*n* = 56 schools]), students ages 9–11 years	Cluster RCT	2004–2005 to 2006–2007 SY	Primary School Free Breakfast Initiative	(1) Diet: healthy food consumption and breakfast skipping (measured using a validated dietary recall questionnaire) (2) Academic Performance: Cognitive tests administered in classrooms	(1) Free breakfast was associated with an ↑ in the number of healthy items at breakfast (0.25 more servings of healthy foods [*p* < 0.01]), with greater improvements observed in lower-income schools. While there was no overall association with breakfast skipping, there was a significant reduction in breakfast skipping among children from lower-income schools and households (*p* < 0.05). (2) No association with cognitive tests.	High
Munday et al. 2017 [81]	New Zealand; one kindergarten class (*n* = 17 students); 2.5 months of exposure to intervention	QE: Pre/post (no control)	2014	Free lunches + educational component	(1) Diet: Foods and nutrients consumed (measure using a 24-h modified dietary recall questionnaire data and a vegetable- and fruit-specific FFQ [teacher and parent-report]) (2) Attendance	(1) Free lunch was associated with ↓ in snack food consumption at school (*p* = 0.015). No association with overall nutrients. (2) No association with attendance.	Very High
Murphy et al. 2011 [71]	Wales; 111 primary schools randomly assigned to free school breakfast (*n* = 55 schools) or control (delayed intervention [*n* = 56 schools]), students ages 9–11 years	Cluster RCT	2004–2005 to 2006–2007 SY	Primary School Free Breakfast Initiative	(1) Diet: healthy food consumption and breakfast skipping (measured using a validated dietary recall questionnaire) (2) Academic Performance: Cognitive tests administered in classrooms	(1) Free breakfast was associated with an ↑ in the number of healthy items at breakfast (0.23 more servings of healthy foods [*p* < 0.01]), with greater improvements observed in lower-income schools. No association with breakfast skipping. (2) No association with cognitive tests.	High
Sabinsky et al. 2018 [83]	Denmark; 4 intervention school and 4 control schools (*n* = 984 students in grades 2–6; students ages 7–13 years	QE: Pre/post (with control)	2008	Free school lunches	Diet: Diet quality (measured using digital photography on 3 consecutive days + a validated Meal Index of dietary Quality (Meal IQ)	Free meals were associated with ↑ dietary quality of the lunch eaten compared with packed lunches (*p* = 0.004), due in part to reductions in saturated fat and snacks and increases in vegetables and fish. When the school meals were not provided for free, selection of these meals was limited and no difference in dietary quality was observed.	Low
Spence et al. 2020 [78]	England; Two primary schools (students age 4–7 years) before and after implementation of UIFSM	QE: Pre/post (no control)	2008–2009 SY and 2017–2018 SY	UIFSM	Diet: Foods and nutrients consumed (measuring using a validated 24-hr food diary on four consecutive days)	UIFSM was associated with ↓ consumption of non-milk extrinsic sugar (i.e., added sugar [mean change −4.6%, *p* < 0.001]) and biscuits (i.e., shortbread cookies [−0.4, *p* < 0.001]) at lunch. The reductions in added sugar were observed in students’ overall diets as well (−3.8%, *p* < 0.001). However, an ↑ in cakes/sweet puddings were observed which were offered with school lunches (after the main meal) daily.	High
Petralia et al. 2016 [86]	Greece; 162 schools provided with free lunches (primary and secondary schools)	QE: Pre/post (no control)	2012–2013 SY	Free lunch (+ nutrition education)	Diet: Food insecurity (measured using the Food Security Survey Module [FSSM]; parent report)	Free school lunch was associated with ↓ food insecurity (decrease from 64.2% of households with food insecurity to 59.1%; *p* < 0.001). The greatest reductions were observed among food insecure households with hunger; each additional month of free school meals was associated with a 13% increase in the odds of not reporting hunger problems (OR 1.13, 95% CI 1.02–1.25).	High
Vik et al. 2019 (BMC Public Health) [76]	Norway; one intervention elementary school with students receiving free school lunch for one year (*n* = 55 students) and one control school (*n* = 109 students); students ages 10–12 years	QE: Pre/post (with control)	2014–2015 SY	Free School Lunches	(1) Diet: Diet quality (measured using validated FFQs, with results used to calculate healthy food scores) (2) BMI: objectively measured at school	(1) Free school lunches were associated with ↑ healthy food scores (F = 10.941, *p* =0.001) after one year of exposure, with the greatest increases among lower-SES students. (2) Free school lunches were associated with ↑ BMI z-scores (F = 10.007, *p* = 0.002) after one year of exposure.	Low
Vik et al. 2019 (BMC Res Notes) [77]	Norway; one intervention elementary school with students receiving free school lunch for one year (*n* = 55 students) and one control school (*n* = 109 students); students ages 10–12 years	QE: Pre/post (with control)	2014–2015 SY	Free School Lunches	Diet: Frequency of consuming meals (measured using a validated questionnaire)	There was no association between free school meals and meal frequency after 1 year of exposure.	Low
Yamaguchi et al. 2018 [80]	Japan; Four municipalities, *n* = 719 elementary school children (ages 6–12 years).	CS	2013	Universal school lunch	Diet: dietary habits (measured using the validated brief diet history questionnaire-10 years old [BDHQ-10]; self-administered)	Universal school lunches were associated with a reduction in SES-related disparities in children’s diets (a reduction in the inequality of vegetable intake by 9.9% and fruit intake by 3.4%)	Low

CS: Cross-Sectional; FFQ: Food Frequency Questionnaire; HEI: Healthy Eating Index; QE: Quasi-Experimental; RCT: Randomized controlled trial. ^1^ Risk of Bias was based on adapted Newcastle–Ottawa Scales (NOS) for cross-sectional and cohort studies (Supplemental Tables S1 and S2).

## 4. Discussion

To our knowledge, this is the first international systematic review of the literature on universal free school meals. This evidence-based review examined the impact of universal free school meals on multiple outcomes for children and schools: (a) meal participation, (b) children’s diet quality and food insecurity, (c) academic performance, (d) attendance, (e) BMI, and (f) school finances. This review found substantial support for a positive association between providing universal free school meals and increased meal participation rates. The current review also found evidence for improvements in diet quality and academic performance when universal free school meal provisions included lunch, whereas the evidence was mixed when only universal free breakfasts were available. Research examining the effect of universal free meals on student attendance was mixed; some studies found that overall attendance improved, while others documented significant improvements specifically among higher-risk populations (e.g., lower-income and/or food insecure). The limited studies examining food security also suggested that universal free school meal provisions were associated with improved food security. Nearly all studies found no adverse associations with BMI. The research examining school finances also suggested that schools in the U.S. with a high percentage of students from low-income households may benefit financially from CEP. Overall, the preponderance of the evidence associates positive outcomes with universal free school meals, particularly provisions that include lunch. These findings outweigh the few studies that raised concerns about possible adverse outcomes.

This literature review found that providing universal free school meals was consistently associated with significant increases in school meal participation. Although increases were generally observed among students previously eligible for free meals [39,48,52,57,61], the largest increases in participation were observed among students not previously eligible for free or reduced-price school meals [39,48,52,58,60,61]. This pattern likely reflects the fact that participation rates are usually lower among students who do not qualify for free or reduced-price school meals, thus providing a greater opportunity for an increase. These findings have several important implications for students. First, the increases in participation among students who were previously eligible for free meals but were not participating suggests that policies to provide universal free school meals may reduce stigma associated with receiving school meals, which can result in more low-income children receiving healthy meals and further reducing food insecurity among a vulnerable population. Prior research has also indicated that universal free school meal policies may be successful by reducing stigma [22,99,100]. Additionally, when universal free school meals are provided, they reach students who may have been eligible based on income but not enrolled for free/reduced price meals. Failure to sign up for free or reduced-price meals may occur due to social stigma, a lack of information, or challenges with enrolling. Universal free meals also reach students who come from households with food insecurity, but which are not eligible for reduced-priced meals due to family incomes slightly above the eligibility threshold (185% of the federal poverty level) [88,101,102,103].

Among the studies examining universal free school lunches (with or without breakfast), positive associations were generally observed with students’ diet quality and academic performance, particularly in the presence of strong nutrition standards that include fruits, vegetables, and/or whole grains. These findings highlight the importance of healthy meal guidelines for schools. Additionally, a government report examining universal free school meals noted that due to the reduction in time spent processing applications for free and reduced-price meals, cafeteria staff time was redirected to improving meal quality, nutrition education, and staff development, which can further the positive influence on students’ dietary behaviors [56]. The limited research examining food security was encouraging, but more high-quality studies are needed to examine if universal free school meals improve food security among students and families. Research has also found that universal free school meals is associated with reductions in students’ behavioral incidents (e.g., fights) and suspensions, which can interfere with academic performance [38,104,105]. Accordingly, investing in universal free school meal programs may have a profound impact on children’s overall health and wellbeing.

In several domains, the results were consistently positive when examining provisions that included universal free lunches, but were mixed when examining schools that only provided universal free breakfasts. The lack of changes observed with universal free breakfast alone may be in part due to the generally low breakfast participation rates observed, despite being universally free. Emerging research examining breakfast in the classroom models suggests that this method of implementing free breakfasts may substantially increase its reach to children [106,107,108]. Future research should examine whether there are additional benefits when children have access to both universal free breakfast in the classroom and universal free lunches. 

While research examining the association between universal free school meals and attendance was mixed, this might partially be explained by the short exposure to universal free school meals in several of the studies. For example, Bartfeld et al. found that no associations with attendance during the first year of implementing CEP, but low attendance rates were significantly reduced after two years of exposure [89]. This may also partially explain why no significant associations were observed among the studies conducted outside of the U.S., which had exposures to free school meals of ≤10 months. Additionally, several studies found that the improvements were only observed among subpopulations, particularly lower-income and/or food insecure students [48,67,89]. This may have important implications for reducing socioeconomic disparities and more long-term studies are warranted. 

Despite concerns about the impact of universal free school meals on BMI, nearly all the studies (6 out of 7) examining BMI found no detrimental impact in terms of increased prevalence of obesity, and in fact, several found a potential reduction in obesity risk associated with universal free meals. This corresponds with several other working papers examining universal free school meals in both U.S. and other OECD countries (i.e., England and Sweden), as well as with peer-reviewed studies examining breakfast in the classroom initiatives and participation in school meal programs more broadly (i.e., means-tested), which have found inverse or no associations with BMI [96,106,109,110,111,112]. For example, Holford and Rabe at the Institute for Social and Economic Research examined UIFSM and found that children receiving free school lunches for a year were 1.2% more likely to be a healthy weight and 0.7% less likely to be obese [111]. Overall, research examining universal free school meals and BMI suggests that in the presence of strong meal standards, providing universal free school meals to students appears to have no adverse impacts on weight and in fact, may reduce the risk of obesity among some populations, although more peer-reviewed research in the U.S. and other countries is warranted. 

School finances were also examined in a limited number of studies, primarily in the U.S. While these studies generally found that school food service budgets may have benefitted from CEP, selection bias may have been an issue. The federal reimbursement rate corresponds to the percentage of students categorically eligible for free meals (the “identified student percentage” [ISP]); therefore, ISP is highly correlated with school/district participation in CEP because schools with a high ISP are more likely to participate and will also have higher federal reimbursement rates [113]. For example, with the current federal reimbursement calculation (which multiplies the ISP by 1.6 to calculate the percentage of meals that will be reimbursed at the rate for free meals), a school with a 40% ISP would receive federal reimbursement that would only cover 64% of all the meals served. Schools actually need an ISP of ≥62.5% in order to be fully reimbursed by the federal government for school meals served with CEP. A policy change to cease the use of ISP and instead fully reimburse schools for all meals served using a national universal free school meal schema would alleviate concerns regarding financial losses to schools. Two additional challenges to school food service finances are that the price charged for full-price meals does not always cover the full cost of the school meal, and that some families fail to pay for the full or reduced-price meals received by their children, resulting in school meal debt. Universal free school meals can help address these issues by shifting the burden of covering school meal costs away from schools and families. In England, the UIFSM was estimated to cost £400 per student annually (roughly USD 550/student) [114]. However, prior research has suggested that universal free school meals may have a positive impact on household finances, particularly among lower income families (many of whom are still above the threshold to qualify for free or reduced-price meals) [115,116]. One study in UK that conducted financial modeling analyses found that families who were in the second and third lowest deciles of income (who were not already eligible for free school meals) benefited the most from universal free school meals [115]. Reducing food insecurity through universal free meals may also help alleviate its concomitant societal costs. For example, in the U.S., food insecurity has been estimated to be associated with over USD 1.2 billion in costs to the health care and education systems [117]. Overall, more research examining the total cost of universal free school meals at the national level is warranted, and this research should account for potential direct and indirect benefits of universal free school meals that may have both short and long-term economic benefits.

Several studies examined differences in outcomes related to universal free school meals by student socio-economic status. Interestingly, many studies found that not only did universal free school meals tend to be associated with positive outcomes among lower-income students─ the population that is typically targeted by free school meal programs─ but higher-income students frequently benefitted as well. For example, higher school meal participation rates were observed among children from both higher and lower-income households in the majority of included studies, and multiple studies found that attendance improved among both lower-income and higher-income populations [48,87,89]. Similarly, two studies found positive associations with academic performance among both lower and higher income students and one found a reduced probability of obesity among higher income students [39,87]. Prior research has also documented improvements in students’ diets after implementation of stronger school meal standards, including among both lower and higher-income students [25,64,118]. This may be in part because many students who are just above eligibility cutoffs (and do not qualify for free or reduced-price meals) also experience food insecurity and therefore may benefit from universal free school meals [119]. Overall, this suggests that universal free school meals may benefit socio-economically diverse student populations and countries that currently limit free school meals to lower-income school districts may want to consider broader policies. 

This study has several limitations. First, the assessment of outcomes varied from one study to another, especially regarding the evaluations of children’s’ diets. For example, while some studies used 24-h recalls, others used food frequency questionnaires or food insecurity questionnaires, and dietary assessments also varied in who completed them (e.g., student self-report or parent-report). While these methods have typically been validated, due to the heterogeneity in outcome measures, a meta-analysis was not possible. The risk of bias also varied from very high to low based on NOS scores, and the results of this review must be interpreted accordingly. However, when examining only the studies with a low risk of bias, the conclusions remained unchanged. Publication bias (i.e., studies with non-significant results are less likely to be published) may have also been an issue, although multiple studies included in the review found no significant associations. The studies reviewed were also conducted in economically developed countries; therefore, future systematic reviews should examine economically less-developed countries, especially as these results suggest that the most vulnerable populations (e.g., food insecure) incur the most benefit from universal free school meals. Lastly, while some studies were randomized, many of relied on natural experiments and therefore schools (and students) were not randomly assigned to receive free meals. However, natural experiments may have better external generalizability due to their implementation in real-world settings. Countries that are considering expanded universal free school meal programs could facilitate evaluations in diverse schools (including those that vary by student SES) through first enabling rigorous designs such as randomized trials before implementation on a wider scale. Such a phased roll-out would enable a fuller understanding of the impact of universal school meals on diverse student populations and school finances. This systematic review was further strengthened by the large number of studies included.

## 5. Conclusions

Overall, this review suggests that universal free school meals benefit students, particularly those who are food-insecure and/or near eligible for free meals in existing means-tested school meal models. The majority of studies in the current review found that universal free school meals were associated with increases in participation and improved diet quality and food security, and conversely, were associated with either no change or improved BMI. Further research is needed regarding the implementation of universal free meal programs and the ways in which optimal participation and benefits can be achieved through such policy changes. In the presence of strong nutrition guidelines, universal free school meals have multiple potential benefits for students and schools, and should be considered by countries not currently with this policy.

## Supplementary Materials

The following are available online at https://www.mdpi.com/2072-6643/13/3/911/s1. Table S1: Summary of School Meal Provisions in the United States, Table S2: Quality Assessment for Cross-Sectional Studies based on the Newcastle Ottawa Quality Assessment Form, Table S3: Quality Assessment of Cohort and Quasi-experimental Studies based on the Newcastle Ottawa Quality Assessment Form.
