# Supplementary material for: Universal School Meals and Associations with Student Participation, Attendance, Academic Performance, Diet Quality, Food Security, and Body Mass Index: A Systematic Review
Source: Nutrients. 2021 Mar 11;13(3):911. doi: 10.3390/nu13030911 (PMC8000006; doi:10.3390/nu13030911)
Supplement: Supplementary file 1 [file nutrients-13-00911-s001.pdf]

Supplemental Table S1. Summary of School Meal Provisions in the United States[1]

Program Type	Description and Eligibility	Administrative Procedures and Reimbursement
CEP	Schools provide free meals to all students regardless of income (universal free meals). Schools (or groups of schools) are eligible to participate if ≥ 40% of students are directly certified as eligible are eligible for free meals.	Students are directly certified (identified) for free school meals through a process that compares the students enrolled in the school with those currently participating in means-tested programs such as SNAP, Medicaid (in selected states), TANF cash assistance, the Food Distribution Program on Indian Reservations (FDPIR), or based on lists of other categorically eligible individuals maintained by school districts, such as foster care. No new eligibility determinations are needed for four consecutive years. No school meal fees are collected and federal reimbursement is based on the percentage of students meeting the direct certification criteria (i.e., "Identified Student Percentage" [ISP]). Reimbursement for meals served equals the ISP multiplied by 1.6).
ERP	Schools provide free meals to students who qualify for free and reduced-price meals but still collect full price meal fees from other students.	School districts process applications and determine eligibility for free and reduced-price meals each school year. Meal fees are collected only for full-price students (reduced-price copays are eliminated) and federal reimbursement is calculated based on the number of free, reduced-price, and full-price meals served. States and/or school district are responsible for the difference in cost for the reduced-price meals not covered by the federal reimbursement rates (which otherwise would have been paid for by the students eligible for reduced-price meals).
Provision 1	Schools can provide free meals to all students, but are not required to (optional universal free meals). Schools are eligible to participate if ≥ 80% of students are eligible for free or reduced-price meals.	School districts conduct free/reduced-priced meal certification from student households every two years (versus annually under the standard school meal program). Schools record numbers of meals served by eligibility type, which is used at the basis for federal reimbursement.
Provision 2	Schools provide free meals to all students regardless of income (universal free meals). There is no minimum percent-eligible requirement for a school to participate.	Schools or districts process applications and determine eligibility for free and reduced-price meals in the first year (base year) of a 4-year period. In the subsequent 3 years, there are no new eligibility determinations. No school meal fees are collected and federal reimbursement is calculated by applying the percentages of free, reduced-price, and full-price meals served during the corresponding month of the base year to the total meal count for the claiming month.
Provision 3	Schools provide free meals to all students regardless of income (universal free meals). There is no minimum percent-eligible requirement for a school to participate.	Schools or districts process applications and determine eligibility for free and reduced-price meals in the year (base year) *preceding* the beginning of the 4-year period. During those four years, there are no new eligibility determinations. No school meal fees are collected and federal reimbursement is calculated based on the number of free, reduced-price, and full price meals served during the base year. In the following years, school food authorities receive the same level of cash subsidies and commodity assistance as they did in the base year, with adjustments for changes in enrollment, number of operating days, and inflation.
Standard (means-tested)	Schools provide free, reduced-price, or full price meals to students based on household income (Free: household income < 130% of the federal poverty level. Reduced-Price: 130-185% of the federal poverty level; Full price: >185% of the federal poverty level).	School districts process household applications and determine student eligibility for free and reduced-price meals each school year (students can also be directly certified for free meals). Meal fees are collected for reduced-price and full-price meals and federal reimbursement is calculated based on the number of free, reduced-price, and full price meals served.

[1]Adapted from Brown et al. 2009 [1]
CEP= Community Eligibility Provision
ERP= Elimination of Reduced-Price Fees Program
SNAP=Supplemental Nutrition Assistance Program (formerly known as food stamps)
TANF= Temporary Assistance for Needy Families

Supplemental Table S2. Quality Assessment for Cross-Sectional Studies based on the Newcastle Ottawa Quality Assessment Form[2]

	Selection				Comparability	Outcome			
**Author**	Representative Sample[1] (+)	Sample Size Justified[2] (+)	Ascertainment of exposure/ risk factor[3] (++)	Non-respondents[4] (+)	Comparability of subjects in different outcome groups; Confounding factors controlled[5] (++)	Assessment of outcome[6] (++)	Statistical Test[7] (+)	Total Score (max 10)	Risk of Bias[8]
**United States**									
Adams et al. 2020 [3]	+	+	++	+	++	++	+	10	Low
Dykstra et al. 2016 [4]		+	++			+	+	5	High
Gross et al. 2019 [5]		+	++		++	+	+	6	High
Khan et al. 2011 [6]		+	++			+		4	High
Poblacion et al. 2017 [7]	+	+	++	+		+	+	7	Low
Soldavini et al. 2019 [8]	+	+	++	+	++	++	+	10	Low
Taylor et al. 2020 [9]	+		++					3	Very High
**Other OECD Countries**									
Asakura et al. 2017 [10]	+	+	++			+		5	High
Gatenby 2011 [11]			++			+		3	Very High
Yamaguchi et al. 2018 [12]	+	+	++		++	+	+	8	Low

OECD= Organization for Economic Co-Operation and Development

[1] **Representative sample**: Evidence the sample is representative of target population (+) *versus* convenience sample or no description.

[2] **Sample size justified**: Justification provided or satisfactory sample size (>100 participants) (+) *versus* no information provided or not satisfactory (<100 participants).

[3] **Ascertainment of exposure/risk factor**: Objective assessment (++), validated non-objective measure (+), *versus* non-objective and non-validated measure.

[4] **Non-respondents**: Proportion of target population recruited attained pre-specified target or basic summary of non-respondent characteristics in sampling frame provided (+) *versus* unsatisfactory recruitment rate or no summary data on non-respondents or no information provided.

[5] **Comparability of subjects in different outcome groups; Confounding factors controlled**: Comparability of subjects in different outcome groups on the basis of design/analyses or analyses adjusted for relevant predictors/risk factors/confounders (++) *versus* information not provided or analyses not adjusted for all relevant confounders/risk factors.

[6] **Assessment of outcome**: Objective assessment (++), validated non-objective measure (+), *versus* non-objective and non-validated measure.

[7] **Statistical test**: Statistical tests used to analyse the data clearly described and appropriate and measures of association presented include confidence intervals and/or probability level (p value) (+) versus statistical tests not appropriate, not described, or incomplete

[8] Total score for the Newcastle–Ottawa Scale (NOS) for assessing the quality of non-randomized studies is attributed to a following categories: very high risk of bias (0–3 NOS points and/or no statistical analyses conducted), high risk of bias (4–6 NOS points), and low risk of bias (7–10 NOS points)

Supplemental Table S3. Quality Assessment of Cohort and Quasi-experimental Studies based on the Newcastle Ottawa Quality Assessment Form [13]

	Selection				Comparability	Outcome				
**Author**	Representative exposed group[1] (+)	Representative non-exposed group[2] (+)	Ascertainment of exposure[3] (+)	Outcome of interest measured at baseline[4] (+)	Comparability of groups; Confounding factors controlled[5] (++)	Assessment of outcome[6] (+)	Adequate follow-up length; ≥1 SY[7] (+)	Adequate subjects retained to follow-up[8] (+)	Total Score (max 9)	Risk of Bias[9]
**United States**										
Bartfeld et al. 2019 [14]	+	+	+	+	++	+	+	+	9	Low
Bartfeld et al. 2020[15]	+	+	+	+	++	+	+	+	9	Low
Bernstein et al. 2004 [16]	+	+	+	+	++	+	+	+	9	Low
Brown 2009 [1]			+	+			+	N/A	3	Very High
Crepinsek et al. 2006 [17]	+	+	+		++	+	+		7	Low
Gordanier et al. 2020 [18]	+	+	+	+	++	+	+	+	9	Low
Kleinman et al. 2002 [19]			+	+		+		+	4	High
Leos-Urbel et al. 2013 [20]	+		+	+	++	+	+	+	8	Low
Logan et al. 2014 [21]		+	+	+	++		+	+	7	Low
McLaughlin et al. 2002 [22]	+	+	+	+ (only for participation, attendance, academic performance)	++	+	+ (only for participation, attendance, academic performance)	+ (only for participation, attendance, academic performance)	6/9[10]	High/ Low[10]

Pokorney et al. 2019 [23]	+	+	+	+	+	+	+	+	8	Low
Ribar et al. 2013 [24]		+	+	+	++	+	+	+	8	Low
Rivas 1994 [25]			+	+	No statistical analyses		+	+	4	Very High
Robinson 1994 [26]			+	+	No statistical analyses		+		3	Very High
Schwartz et al. 2020 [27]	+		+	+	++	+	+	+	8	Low
Tan et al. 2020 [28]	+	+	+		++	+	+	N/A	7	Low
Turner et al. 2019 [29]	+	+	+	+	++	+	+	+	9	Low
Wahlstrom et al. 1999 [30]		+	+	+	No statistical analyses	+	+	+	6	Very High
**Other OECD Countries**										
Andersen et al. 2014 [31]	+	+	+	+	++	+		+	8	Low
Ask et al. 2006 [32]			+	+				+	3	Very High
Ask et al. 2010 [33]			+	+				+	3	Very High
Bartelink et al. 2019[34]	+	+	+	+	++	+	+	+	9	Low
Dalma et al. 2020 [35]	+	+	+	+	++	+			7	Low
Holford 2015 [36]	+	+	+	+	+	+	+	+	8	Low
IlløKken et al. 2017 [37]			+	+		+		+	4	High
Jenkins et al. 2015 [38]	+	+	+	+		+	+		6	High

Laursen et al. 2015 [39]	+	+	+	+	++	+		+	8	Low
MacLardie et al. 2008 [40]	+		+	+	No statistical analyses	+		+	5	Very High
Mhurchu et al. 2012 [41]		+	+	+	++	+		+	7	Low
Moore et al. 2014 [42]	+	+	+	+		+	+		6	High
Munday et al. 2017 [43]			+	+				+	3	Very High
Murphy et al. 2011[44]	+	+	+	+	+	+	+		7	High
Petralia et al. 2016 [45]	+		+	+	+	+			5	High
Sabinsky et al. 2018 [46]	+	+	+	+	++	+		+	8	Low
Spence et al. 2020 [47]			+	+	+	+	+		5	High
Vik et al. 2019 (BMC Public Health) [48]		+	+	+	+	+	+	+	7	Low
Vik et al. 2019 (BMC Res Notes) [49]		+	+	+	+	+		+	7	Low

OECD= Organization for Economic Co-Operation and Development

[1] **Representative exposed group**: Evidence the sample in the exposed group is representative of target population (+) *versus* convenience sample or no description.

[2] **Representative non-exposed group**: Evidence the sample in the non-exposed group is drawn from the same community as the exposed group (+) *versus* drawn from a different source or no description.

[3] ***Ascertainment of exposure/risk factor**: Objective assessment (++), validated non-objective measure (+), *versus* non-objective and non-validated measure.

[4] ***Outcome of interest measured at baseline**: Baseline measurements collected (+) *versus* no baseline assessments.

[5] ***Comparability of groups; Confounding factors controlled**: Comparability of subjects in different outcome groups and analyses adjusted for relevant predictors/risk factors/confounders (++), adjusted for some but not all relevant confounders or statistical analyses did not account for clustered design (+), *versus* information not provided or analyses not adjusted for relevant confounders/risk factors.

[6] ***Assessment of outcome**: Objective assessment (++), validated non-objective measure (+), *versus* non-objective and non-validated measure.

[7] **Adequate follow-up length; ≥1 SY**: Participants are followed-up for at least one school year (+) *versus* follow-up is less than one school year

[8] **Adequate subjects retained to follow-up**: Loss to follow-up was ≤15% (+) *versus* >15% loss to follow-up among participants

[9] Total score for the Newcastle–Ottawa Scale (NOS) for assessing the quality of cohort studies is attributed to a following categories: very high risk of bias (0–3 NOS points and/or no statistical analyses conducted), high risk of bias (4–6 NOS points), and low risk of bias (7–9 NOS points)
[10]Total Score/Risk of Bias for McLaughlin et al was 6 (high) for diet, BMI, and finances, and 9 (low) for participation, attendance, and academic performance.
*Denotes minimally modified from the original NOS for assessing the quality of cohort studies

References

1. Brown, K. School Meal Programs: Experiences of the States and Districts That Eliminated Reduced-Price Fees. Report to the Chairman, Committee on Education and Labor, House of Representatives. GAO-09-584. *US Government Accountability Office* **2009**.
2. Wells, G.A.; Shea, B.; O'Connell, D.a.; Peterson, J.; Welch, V.; Losos, M.; Tugwell, P. The Newcastle-Ottawa Scale (NOS) for assessing the quality of nonrandomised studies in meta-analyses. Oxford: 2000.
3. Adams, E.L.; Raynor, H.A.; Thornton, L.M.; Mazzeo, S.E.; Bean, M.K. Using digital imagery to quantify students' added sugar intake at lunch in Title I schools with universal free meals. *Preventive Medicine Reports* **2020**, *20*, 101253.
4. Dykstra, H.; Davey, A.; Fisher, J.O.; Polonsky, H.; Sherman, S.; Abel, M.L.; Dale, L.C.; Foster, G.D.; Bauer, K.W. Breakfast-skipping and selecting low-nutritional-quality foods for breakfast are common among low-income urban children, regardless of food security status. *The Journal of nutrition* **2016**, *146*, 630-636.
5. Gross, S.M.; Kelley, T.L.; Augustyn, M.; Wilson, M.J.; Bassarab, K.; Palmer, A. Household Food Security Status of Families with Children Attending Schools that Participate in the Community Eligibility Provision (CEP) and Those with Children Attending Schools that are CEP-Eligible, but Not Participating. *Journal of Hunger & Environmental Nutrition* **2019**, 1-16.
6. Khan, S.; Pinckney, R.G.; Keeney, D.; Frankowski, B.; Carney, J.K. Prevalence of food insecurity and utilization of food assistance program: an exploratory survey of a Vermont middle school. *Journal of School Health* **2011**, *81*, 15-20.
7. Poblacion, A.; Cook, J.; Ettinger de Cuba, S.; Bovell, A.; Sheward, R.; Pasquariello, J.; Cutts, D. Can food insecurity be reduced in the United States by improving SNAP, WIC, and the community eligibility provision? *World Medical & Health Policy* **2017**, *9*, 435-455.
8. Soldavini, J.; Ammerman, A.S. Serving breakfast free to all students and type of breakfast serving model are associated with participation in the School Breakfast Program. *Journal of the Academy of Nutrition and Dietetics* **2019**, *119*, 1142-1149.
9. Taylor, J.; Garnett, B.; Horton, M.A.; Farineau, G. Universal Free School Meal Programs in Vermont Show Multi-domain Benefits. *Journal of Hunger & Environmental Nutrition* **2020**, 1-14.
10. Asakura, K.; Sasaki, S. School lunches in Japan: their contribution to healthier nutrient intake among elementary-school and junior high-school children. *Public health nutrition* **2017**, *20*, 1523-1533.
11. Gatenby, L. Children's nutritional intake as part of the Eat Well Do Well scheme in Kingston-upon-Hull–a pilot study. *Nutrition Bulletin* **2011**, *36*, 87-94.
12. Yamaguchi, M.; Kondo, N.; Hashimoto, H. Universal school lunch programme closes a socioeconomic gap in fruit and vegetable intakes among school children in Japan. *The European Journal of Public Health* **2018**, *28*, 636-641.
13. Wells, G.; Shea, B.; O'Connell, D.; Peterson, J.; Welch, V.; Losos, M.; Tugwell, P. Newcastle-Ottawa quality assessment scale cohort studies. 2014.
14. Bartfeld, J.S.; Berger, L.; Men, F.; Chen, Y. Access to the school breakfast program is associated with higher attendance and test scores among elementary school students. *The Journal of nutrition* **2019**, *149*, 336-343.
15. Bartfeld, J.S.; Berger, L.; Men, F. Universal Access to Free School Meals through the Community Eligibility Provision Is Associated with Better Attendance for Low-Income Elementary School Students in Wisconsin. *Journal of the Academy of Nutrition and Dietetics* **2020**, *120*, 210-218.
16. Bernstein, L.S.; McLaughlin, J.E.; Crepinsek, M.K.; Daft, L.M. Evaluation of the School Breakfast Program Pilot Project: Final Report. Special Nutrition Programs. Report Number CN-04-SBP. Nutrition Assistance Program Report Series. *US Department of Agriculture* **2004**.
17. Crepinsek, M.K.; Singh, A.; Bernstein, L.S.; McLaughlin, J.E. Dietary effects of universal-free school breakfast: findings from the evaluation of the school breakfast program pilot project. *Journal of the American Dietetic Association* **2006**, *106*, 1796-1803.
18. Gordanier, J.; Ozturk, O.; Williams, B.; Zhan, C. Free lunch for all! the effect of the community eligibility provision on academic outcomes. *Economics of Education Review* **2020**, *77*, 101999.

19. Kleinman, R.E.; Hall, S.; Green, H.; Korzec-Ramirez, D.; Patton, K.; Pagano, M.E.; Murphy, J.M. Diet, breakfast, and academic performance in children. *Annals of Nutrition and Metabolism* **2002**, *46*, 24-30.
20. Leos-Urbel, J.; Schwartz, A.E.; Weinstein, M.; Corcoran, S. Not just for poor kids: The impact of universal free school breakfast on meal participation and student outcomes. *Economics of education review* **2013**, *36*, 88-107.
21. Logan, C.W. *Community eligibility provision evaluation*; United States Department of Agriculture, Food and Nutrition Service, Office …: 2014.
22. McLaughlin, J.E.; Bernstein, L.S.; Crepinsek, M.K.; Daft, L.M.; Murphy, J.M. Evaluation of the School Breakfast Program Pilot Project: Findings from the First Year of Implementation. Nutrition Assistance Program Report Series. **2002**.
23. Pokorney, P.E.; Chandran, A.; Long, M.W. Impact of the Community Eligibility Provision on meal counts and participation in Pennsylvania and Maryland National School Lunch Programs. *Public health nutrition* **2019**, *22*, 3281-3287.
24. Ribar, D.C.; Haldeman, L.A. Changes in meal participation, attendance, and test scores associated with the availability of universal free school breakfasts. *Social Service Review* **2013**, *87*, 354-385.
25. Rivas, D. Everyone Eats for Free--Piloting Provision 2. *School Business Affairs* **1994**, *61*.
26. Robinson, R.A. Food Assistance: Early Results of USDA's No-Fee School Meal Pilot Program. Report to U.S. Government Accountability Office (GAO). 1994.
27. Schwartz, A.E.; Rothbart, M.W. Let them eat lunch: The impact of universal free meals on student performance. *Journal of Policy Analysis and Management* **2020**, *39*, 376-410.
28. Tan, M.L.; Laraia, B.; Madsen, K.A.; Johnson, R.C.; Ritchie, L. Community Eligibility Provision and School Meal Participation among Student Subgroups. *Journal of School Health* **2020**, *90*, 802-811.
29. Turner, L.; Guthrie, J.F.; Ralston, K. Community eligibility and other provisions for universal free meals at school: impact on student breakfast and lunch participation in California public schools. *Translational behavioral medicine* **2019**, *9*, 931-941.
30. Wahlstrom, K.L.; Begalle, M.S. More than test scores: results of the Universal School Breakfast Pilot in Minnesota. *Topics in clinical nutrition* **1999**, *15*, 17-29.
31. Andersen, R.; Biltoft-Jensen, A.; Christensen, T.; Andersen, E.W.; Ege, M.; Thorsen, A.V.; Dalskov, S.-M.; Damsgaard, C.T.; Astrup, A.; Michaelsen, K.F. Dietary effects of introducing school meals based on the New Nordic Diet–a randomised controlled trial in Danish children. The OPUS School Meal Study. *British journal of nutrition* **2014**, *111*, 1967-1976.
32. Ask, A.S.; Hernes, S.; Aarek, I.; Johannessen, G.; Haugen, M. Changes in dietary pattern in 15 year old adolescents following a 4 month dietary intervention with school breakfast–a pilot study. *Nutrition Journal* **2006**, *5*, 33.
33. Ask, A.S.; Hernes, S.; Aarek, I.; Vik, F.; Brodahl, C.; Haugen, M. Serving of free school lunch to secondary-school pupils–a pilot study with health implications. *Public health nutrition* **2010**, *13*, 238-244.
34. Bartelink, N.H.; van Assema, P.; Kremers, S.P.; Savelberg, H.H.; Oosterhoff, M.; Willeboordse, M.; van Schayck, O.C.; Winkens, B.; Jansen, M.W. Can the Healthy Primary School of the Future offer perspective in the ongoing obesity epidemic in young children? A Dutch quasi-experimental study. *BMJ open* **2019**, *9*.
35. Dalma, A.; Petralias, A.; Tsiampalis, T.; Nikolakopoulos, S.; Veloudaki, A.; Kastorini, C.-M.; Papadimitriou, E.; Zota, D.; Linos, A. Effectiveness of a school food aid programme in improving household food insecurity; a cluster randomized trial. *European Journal of Public Health* **2020**, *30*, 171-178.
36. Holford, A. Take-up of Free School Meals: Price Effects and Peer Effects. *Economica* **2015**, *82*, 976-993.
37. IlløKken, K.E.; Bere, E.; Øverby, N.C.; Høiland, R.; Petersson, K.O.; Vik, F.N. Intervention study on school meal habits in Norwegian 10–12-year-old children. *Scandinavian Journal of Public Health* **2017**, *45*, 485-491.
38. Jenkins, K.T.; Benton, D.; Tapper, K.; Murphy, S.; Moore, L. A cross-sectional observational study of the nutritional intake of UK primary school children from deprived and non-deprived backgrounds: implications for school breakfast schemes. *International Journal of Behavioral Nutrition and Physical Activity* **2015**, *12*, 86.

39. Laursen, R.P.; Lauritzen, L.; Ritz, C.; Dyssegaard, C.; Astrup, A.; Michaelsen, K.F.; Damsgaard, C.T. Do healthy school meals affect illness, allergies and school attendance in 8-to 11-year-old children? A cluster-randomised controlled study. *European Journal of Clinical Nutrition* **2015**, *69*, 626-631.
40. MacLardie, J.; Martin, C.; Murray, L.; Sewel, K. Evaluation of the free school meals trial for P1 to P3 pupils. Edinburgh, Scottish Government Social Research. 2008.
41. Mhurchu, C.N.; Gorton, D.; Turley, M.; Jiang, Y.; Michie, J.; Maddison, R.; Hattie, J. Effects of a free school breakfast programme on children's attendance, academic achievement and short-term hunger: results from a stepped-wedge, cluster randomised controlled trial. *Journal of Epidemiology & Community Health* **2013**, *67*, 257-264.
42. Moore, G.F.; Murphy, S.; Chaplin, K.; Lyons, R.A.; Atkinson, M.; Moore, L. Impacts of the Primary School Free Breakfast Initiative on socio-economic inequalities in breakfast consumption among 9–11-year-old schoolchildren in Wales. *Public Health Nutrition* **2014**, *17*, 1280-1289.
43. Munday, K.; Wilson, M. Implementing a health and wellbeing programme for children in early childhood: A preliminary study. *Nutrients* **2017**, *9*, 1031.
44. Murphy, S.; Moore, G.; Tapper, K.; Lynch, R.; Clarke, R.; Raisanen, L.; Desousa, C.; Moore, L. Free healthy breakfasts in primary schools: a cluster randomised controlled trial of a policy intervention in Wales, UK. *Public health nutrition* **2011**, *14*, 219-226.
45. Petralias, A.; Papadimitriou, E.; Riza, E.; Karagas, M.R.; Zagouras, A.B.; Linos, A.; Team, D.P.R. The impact of a school food aid program on household food insecurity. *The European Journal of Public Health* **2016**, *26*, 290-296.
46. Sabinsky, M.S.; Toft, U.; Sommer, H.M.; Tetens, I. Effect of implementing school meals compared with packed lunches on quality of dietary intake among children aged 7–13 years. *Journal of nutritional science* **2019**, *8*.
47. Spence, S.; Matthews, J.; McSweeney, L.; Rowland, M.; Orango, P.; Adamson, A. Implementation of Universal Infant Free School Meals: a pilot study in NE England exploring the impact on Key Stage 1 pupil's dietary intake. *Public Health Nutrition* **2020**, 1-22.
48. Vik, F.N.; Van Lippevelde, W.; Øverby, N.C. Free school meals as an approach to reduce health inequalities among 10–12-year-old Norwegian children. *BMC public health* **2019**, *19*, 951.
49. Vik, F.N.; Næss, I.K.; Heslien, K.E.; Øverby, N.C. Possible effects of a free, healthy school meal on overall meal frequency among 10–12-year-olds in Norway: the School Meal Project. *BMC Research Notes* **2019**, *12*, 382.
